# High expression of apoptosis protein (Api-5) in chemoresistant triple-negative breast cancers: an innovative target

**DOI:** 10.18632/oncotarget.27312

**Published:** 2019-11-12

**Authors:** Guilhem Bousquet, Jean-Paul Feugeas, Yuchen Gu, Christophe Leboeuf, Morad El Bouchtaoui, He Lu, Marc Espié, Anne Janin, Melanie Di Benedetto

**Affiliations:** ^1^Université Paris Diderot, Sorbonne Paris Cité, Laboratoire de Pathologie, UMR-S 1165, F-75010, Paris, France; ^2^INSERM, U942, F-75010, Paris, France; ^3^Université Paris 13, Sorbonne Paris Cite, F-93000, Villetaneuse, France; ^4^INSERM, U1137-Paris, F-75018, France; ^5^AP-HP, Hôpital Saint-Louis, Centre des Maladies du Sein, F-75010, Université Paris Diderot, Sorbonne Paris Cité, INSERM CNRS UMR7212, Paris, France; ^6^AP-HP, Hôpital Avicenne, Medical Oncology, F-93000, Bobigny, France; ^7^AP-HP, Hôpital Saint-Louis, Laboratoire de Pathologie, F-75010, Paris, France

**Keywords:** triple-negative breast cancer, chemotherapy resistance, apoptosis-inhibitor-5, peptide, anti-angiogenic therapy

## Abstract

Anti-apoptotic protein-5 (API-5) is a survival protein interacting with the protein acinus, preventing its cleavage by caspase-3 and thus inhibiting apoptosis. We studied the effect of targeting API-5 in chemoresistant triple negative breast cancers (TNBCs), to reverse chemoresistance.

78 TNBC biopsies from patients with different responses to chemotherapy were analysed for API-5 expression before any treatment. Further studies on API-5 expression and inhibition were performed on patient-derived TNBC xenografts, one highly sensitive to chemotherapies (XBC-S) and the other resistant to most tested drugs (XBC-R). *In situ* assessments of necrosis, cell proliferation, angiogenesis, and apoptosis in response to anti-API-5 peptide were performed on the TNBC xenografts.

Clinical analyses of the 78 TNBC biopsies revealed that API-5 was more markedly expressed in endothelial cells before any treatment among patients with chemoresistant TNBC, and this was associated with greater micro-vessel density. A transcriptomic analysis of xenografted tumors showed an involvement of anti-apoptotic genes in the XBC-R model, and *API-5* expression was higher in XBC-R endothelial cells. *API-5* expression was also correlated with hypoxic stress conditions both *in vitro* and *in vivo*. 28 days of anti-API-5 peptide efficiently inhibited the XBC-R xenograft via caspase-3 apoptosis. This inhibition was associated with major inhibition of angiogenesis associated with necrosis and apoptosis.

API-5 protein could be a valid therapeutic target in chemoresistant metastatic TNBC.

## INTRODUCTION

Apoptosis Inhibitor-5 (API-5) also called Anti-Apoptosis Clone 11 (AAC-11) is a 58-kDa nuclear protein highly conserved across species [1]. API-5 contains two acid domains, three overlapping heptad repeats of hydrophobic amino acid motifs, a leucine zipper (LZ) and a nuclear localization sequence (Supplementary Figure 1A). API-5 was originally identified as an anti-apoptotic protein in mouse fibroblasts and in human cervical carcinoma cell lines, in which API-5 significantly enhanced cell survival after serum deprivation or ultraviolet sensitization [1–4]. *In vitro*, a decrease in API-5 expression sensitized human cancer cell lines to apoptosis, and increased their sensitivity to anticancer drugs [5–7]. In human osteocarcinoma cells, *API-5* promotes survival through the inhibition of an E2 promoter-binding factor [E2F1] [6], and the integrity of the leucine zipper domain is required for the anti-apoptotic functions of API-5 [7]. API-5 interacts with the protein acinus, preventing its cleavage by caspase-3, and thus inhibiting the generation of an active P17 fragment, which in turn leads to DNA fragmentation [7], (Supplementary Figure 1B).

Previous authors engineered an anti-API5 peptide composed of the LZ domain fused with the *Antennapedia/*Penetrating protein domain (Supplementary Figure 1A) since the integrity of this domain is essential for the function of API-5 [7, 8] This LZ peptide is able to penetrate cells *in vitro*, and to cancel the API-5/acinus interaction.

Triple-negative breast cancer (TNBC) accounts for 10% of breast cancers, and occurs mainly in young women. Resistance to treatments results in the development of metastasis and a median survival of under 15 months [9, 10]. The standard treatment for metastatic TNBC is a combination of cytotoxic drugs. There is no available targeted therapy because of the absence of any targetable receptors. Most cytotoxic drugs act through the activation of cell death pathways, and resistance may be linked to alterations in genes regulating apoptosis [11–15]. This provides potential therapeutic applications for the use of anti-API-5 peptide in chemotherapy-resistant TNBC.

The use of pre-clinical models is a key component of translational research in oncology. For four decades, xenograft models of human cancer cell lines injected subcutaneously in immuno-compromised mice have been widely used, but with disappointing results in predicting the clinical benefit of a new drug [16]. One major explanation is that their durable *in vitro* development results from genetic stress and changes that do not reflect the carcinogenesis process in a patient [17]. Patient-derived xenografts are more relevant pre-clinical models [18, 19]. We have already established patient-derived TNBC xenografts and have shown that they are relevant pharmacological tools for personalized resort treatment among women with metastatic TNBC [18–20]. In this pre-clinical study, we studied the expression of API-5 in patients with chemotherapy-resistant TNBC and the inhibition of API-5 in a resistant TNBC xenograft model.

## RESULTS

### API-5 expression is higher in patient-derived endothelial cells of chemoresistant TNBC

Since API-5 is involved in resistance to apoptosis [1], we postulated that TNBC chemoresistance was partly due to tumor resistance to apoptosis and thus to API-5 expression before any treatment.

TNBC frozen biopsies were obtained from 78 women with locally advanced TNBC, before any treatment. Regarding response to chemotherapy before surgery, we identified two groups of patients, with and without pathological complete response (pCR), respectively 20 and 58 women.

**Figure 1:**
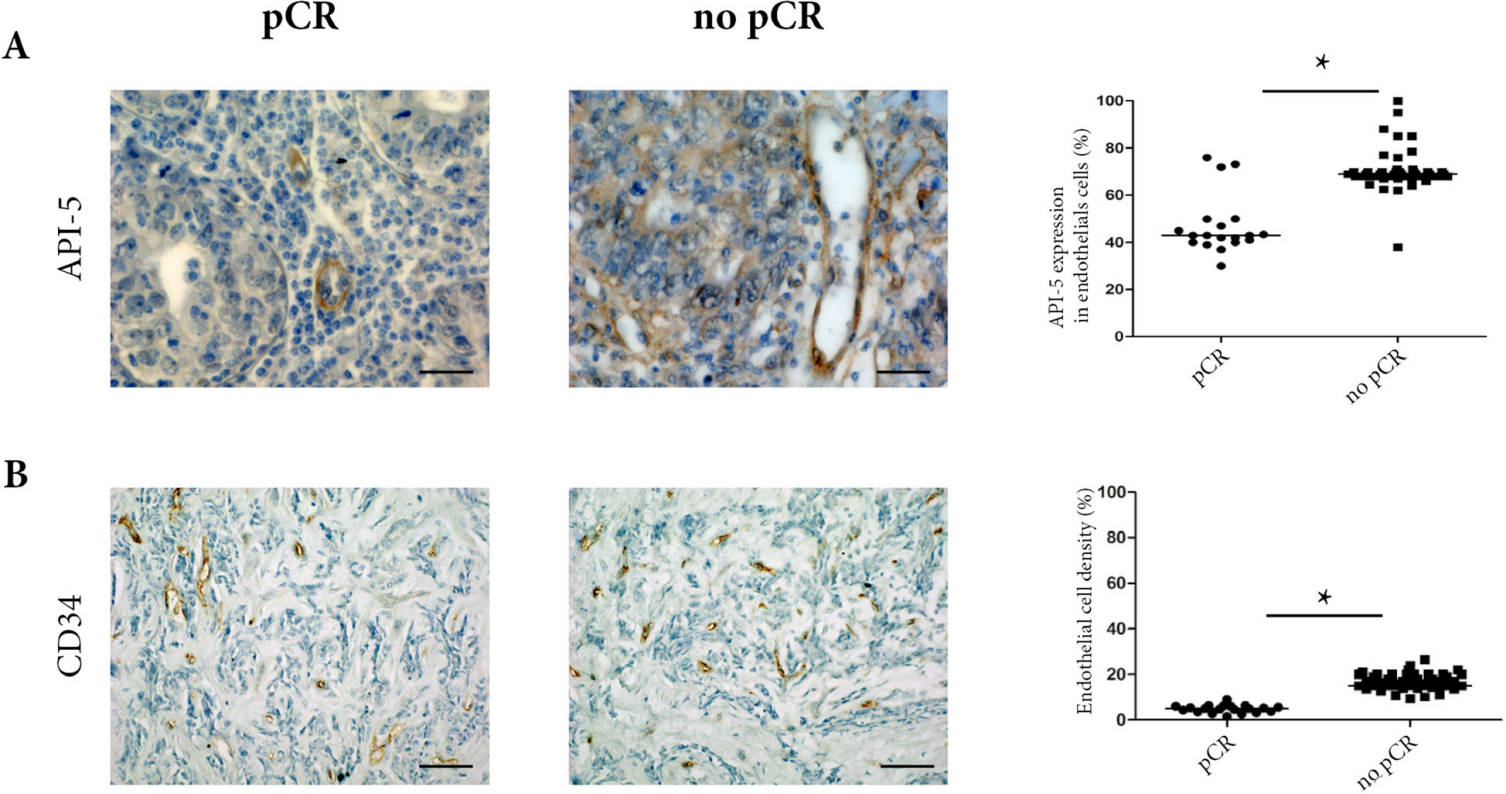
Clinical analyses of 78 TNBC biopsies from pCR and non-pCR patients before any treatment. (**A**) API-5 immunostaining at x400 magnification in pCR and non pCR. The percentage of API-5 expression (right panel) in endothelial cells confirms high levels of API-5 expression in non-pCR biopsies. For the scoring, the percentage of positive cells in 100 cells was determined, and results were expressed as mean ± SEM. (**B**) Endothelial cells were labelled using CD34 antibody in pCR and non-pCR biopsies (left panel) at x200 magnification. The percentage of positive cells and vascular densities were determined as described in materials and methods (right panel).

When we assessed API-5 expression using simple peroxidase immunostaining, API-5 was mainly expressed in tumor endothelial cells. Expression was detected in both nuclear and cytoplasm of endothelial cells. In addition, when we counted the percentage of tumor endothelial cells expressing API-5 using immunohistochemistry, we found a significantly higher percentage in non-pCR patients (40% pCR vs 70% non-pCR, *p* = 0.01, Figure 1A). Then we assessed tumor microvessel density and found that it was significantly higher in non-pCR patients (7% pCR versus 17% non-pCR, *p* < 0.05, Figure 1B). Total RT-PCR did not enable a sufficient amount of API-5 expression to be detected in endothelial cells to show any significant difference in expression between pCR and non-pCR patients (data not shown).

### Assessment of *in vivo* toxicity of anti-API-5 peptide and determination of optimal therapeutic dose in preclinical mouse models

In order to block API-5 expression by a peptide in patient-derived TNBC xenograft models, the maximum tolerated dose of anti-API-5 peptide was assessed in nude mice without any tumor. We tested a range of concentrations from 0.8 to 4.8 mg/kg. No toxic death was observed over the time of treatment. We systematically assessed *in situ* toxicity on all organs after 28 days of treatment. Toxic effects were mainly observed at the highest dose of 4.8 mg/kg, with a diffuse cytoplasmic clarification of hepatocytes. This was associated with a mild intra-alveolar edema in the lung. No other toxic effect was found (Supplementary Figure 2). We therefore chose a dose of 2.4 mg/kg for the treatment of the xenografted mice.

### Two opposite pharmacological models of patient-derived TNBC xenografts

**Figure 2:**
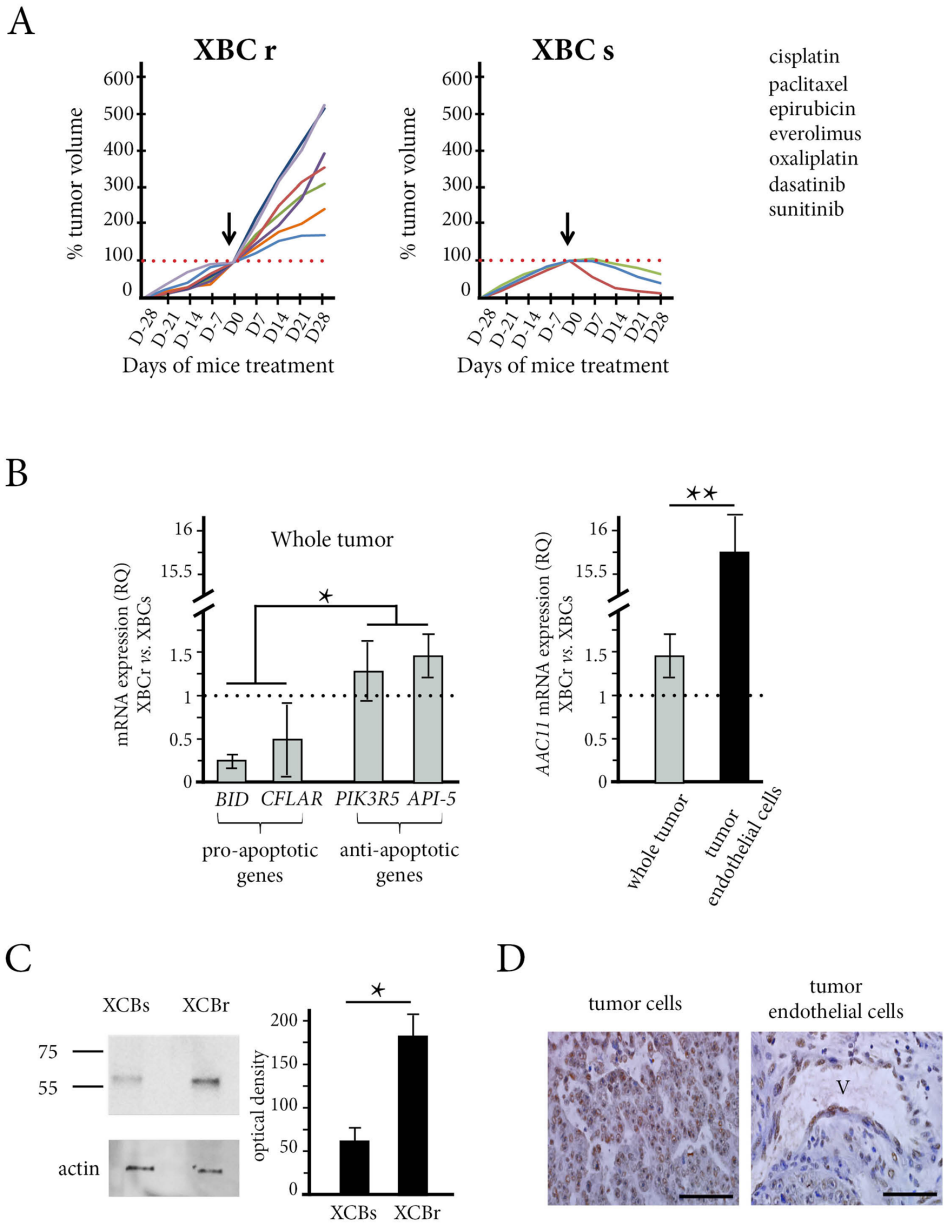
The *in vivo* effect of chemotherapy and API-5 expression in the patient xenograft model obtained from resistant tumors (XBC-R right panel) and sensitive tumors (XBC-S, left panel). (**A**) The mice (*n* = 5 in each group) were treated with the different treatments (see Supplementary Table 1) and the tumors were measured every week from 4 weeks before the treatment started (arrows at day 0) to 4 weeks after the start of treatment. Tumor volumes showed that the XBC-R model was resistant to the different chemotherapeutic drugs tested whereas the XBC-S model was sensitive. (**B**) The relative expression of *BID*, *PIK3R5*, *API-5* and *c-FLAR/cFLIP* (left panel) by XBC-R tumors versus XBC-S tumors showed high levels of expression of anti-apoptotic genes. Tumor analysis (*n* = 10) of API-5 expression in tumors and tumor endothelial cells (right panel) showed a more than 15-fold increase in API-5 expression in XBC-R versus XBC-S in tumor endothelial cells. The results are expressed as 2^[-∆∆CT].^^*^ and ^**^ *p* XBC-S versus XBC-R <0.05 and 0.01 respectively. (**C**) Western Blotting of API-5 expression in XBC-S and XBC-R confirms high levels of expression in XBC-R. beta-actin was used to measure total protein. (**D**) API-5 immunostaining of XBC-R at x200 (left panel) and x400 (right panel) magnification confirms high levels of expression of API-5 in XBC-R.

We established two TNBC xenograft models with opposite profiles of sensitivity to drugs (Figure 2A). We tested three cytotoxic drugs currently used for the treatment of metastatic TNBC: cisplatin, paclitaxel and epirubicin (Supplementary Table 1). One model was resistant to the three drugs with growth inhibition coefficients of 1.07, 1.57 and 0.10 for cisplatin, paclitaxel and epirubicin respectively (Figure 2A, left panel and Table 1). We then tested four other chemotherapeutic agents on the same xenograft model, including another platinum compound (oxaliplatin), two anti-angiogenic drugs (everolimus and sunitinib) and a Src/Abl inhibitor (dasatinib). None of these drugs induced an anti-tumor effect. We called this TNBC xenograft model XBC-R.

**Table 1:** Growth inhibition coefficient for drugs tested in XBC-R model

Drug	Growth inhibition coefficient
Cisplatin	1.07
Paclitaxel	1.57
Epirubicin	0.10
Everolimus	3.22
Oxaliplatin	0.23
Dasatinib	2.54
**anti-API-5 peptide**	**–0.60**

In contrast, the other TNBC xenograft model was sensitive to cisplatin, paclitaxel and epirubicin with negative growth inhibition coefficients (Figure 2A, right panel and Table 2). We called this TNBC xenograft model XBC-S.

**Table 2:** Growth inhibition coefficient for drugs tested in XBC-S model

Drug	Growth inhibition coefficient
Cisplatin	–1.43
Paclitaxel	–6.34
Epirubicin	–1.50
**AAC-11 peptide**	**–0.80**

### Differential apoptosis pathways in XBC-R and XBC-S xenograft models

Using transcriptomic analyses, we found that the XBC-S xenograft model derived from a tumor classified as BL1 according to Lehmann’s molecular sub-classification of TNBC [21] The XBC-R xenograft model derived from a tumor classified as stem-like (MSL). We confirmed that the XBC-S and XBC-R tumor xenografts were also classified as BL1 and MSL respectively.

Since we detected more API-5 in patient without complete response to chemotherapy, we examined if some global differences in apoptosis could exist between the two models. We then compared the transcriptomic profiles of XBC-R and XBC-S tumors. Using a *p*-value of 0.05 or under, and a fold greater than 2 for overexpression and below 2 for underexpression, we found 723 genes that were differentially expressed (Using the Database for Annotation, Visualization and Integrated Discoverymethod (DAVID)). The complete transcriptomic data were published in our previous work [22]. For pathway analyses, we focused on the KEGG pathway (Kyoto Encyclopedia of Genes and Genomes) and identified 11 pathways, including an apoptosis pathway (Supplementary Table 2, Supplementary Figure 4). In particular, the XBC-S chemo-sensitive model overexpressed pro-apoptotic genes (*BID* and *C-FLAR*) whereas the XBC-R model overexpressed anti-apoptotic genes (*PIK3R5, TNF, IL1B, NFKB, IL3R*). Since we hypothesized that XBC resistance/sensitivity were linked to a particular apoptosis status, we used RT-qPCR to validate these genes. The results confirmed that *BID* and *CFLAR/C-FLIP* mRNA were significantly under-expressed in XBC-R compared to XBC-S (RQ = 0.25 and 0.52 respectively, *p* < 0.05) and that *PIK3R5* mRNA was significantly overexpressed in XBC-R (RQ = 1.33, *p* < 0.05) (Figure 2B, left panel).

### API-5 expression in XBC-R and XBC-S TNBC xenograft models

When we assessed human *API-5* mRNA expression in tumor cells (*n* = 5 for each) of the XBC-S and XBC-R models, we found that *API-5* was significantly over-expressed in XBC-R tumor xenografts (RQ = 1.4, *p* < 0.05) (Figure 2B, left panel). We then wondered if there was also a differential expression of *API-5* in murine tumor endothelial cells of the XBC-R and XBC-S tumor xenografts. CD105 expression was known to be up-regulated in actively proliferating tumor endothelial cells [23]. Using a murine probe, we assessed *API-5* mRNA expression in CD105-positive tumor endothelial cells sorted from the two xenograft models. Strikingly, *API-5* expression was even higher in endothelial cells sorted from XBC-R compared to those from XBC-S xenografts (RQ = 14, *p* < 0.05) (Figure 2B, right panel).

API-5 protein expression was assessed in XBC-R tumors by immunochemistry. We showed that both tumor cells and endothelial cells expressed API-5 (Figure 2C). Since the quantity of total proteins in tumor endothelial cells was insufficient for Western Blotting, we analysed the total proteins of the whole tumor. We found a significantly larger quantity of API-5 (3 times more) (band at 58KD as indicated by the manufacturer) in XBC-R compared to XBC-S xenografts (*p* < 0.05, Figure 2D).

Overall, our results showed higher levels of expression of API-5 in chemoresistant TNBC xenografts, for both protein and mRNA, particularly in tumor endothelial cells.

### Anti-API-5 peptide inhibits tumor growth in chemoresistant TNBC xenografts

**Figure 3:**
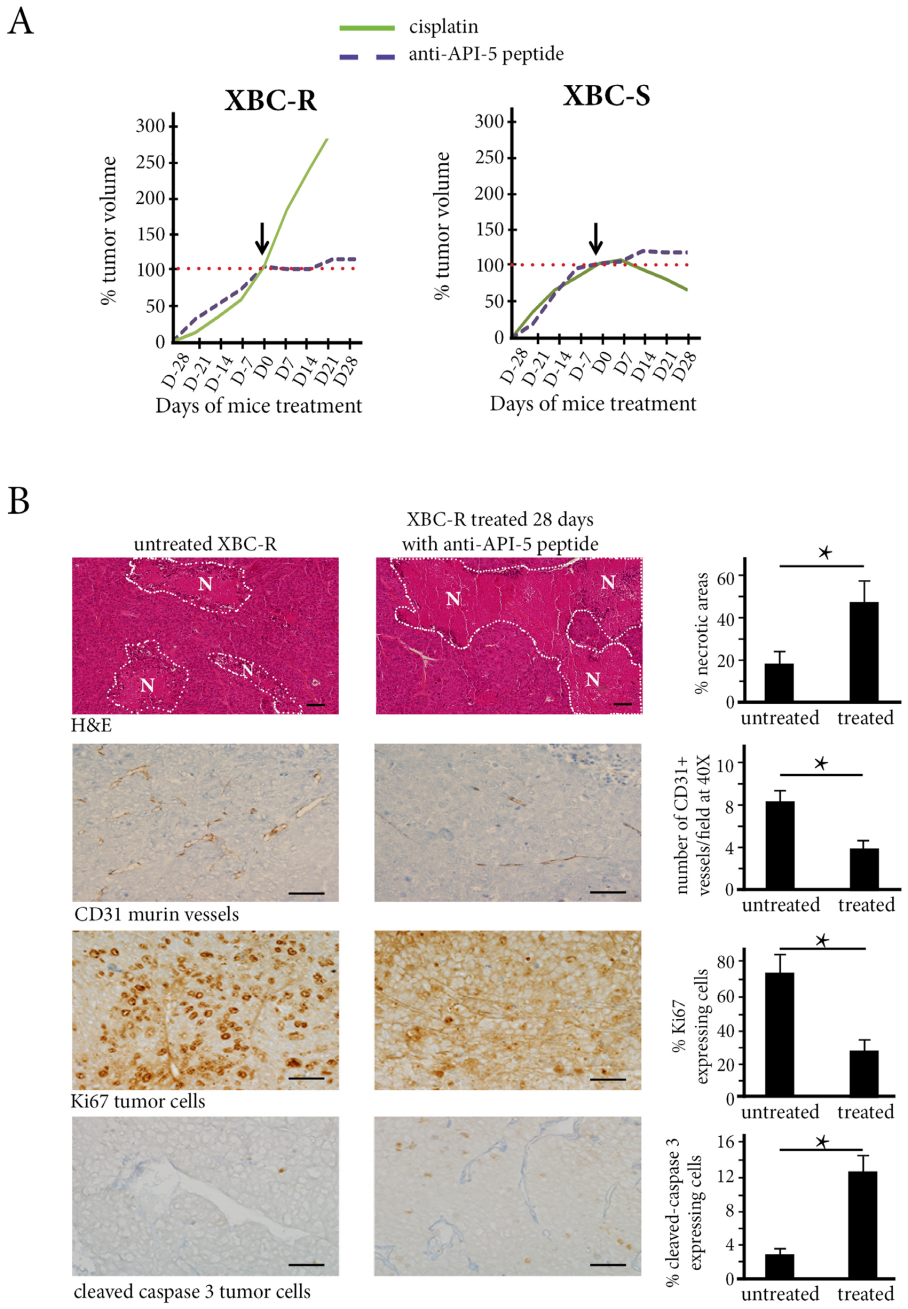
The *in vivo* effect of Cisplatin and API-5 peptide in XBC-R (left panel) and XBC-S (right panel). (**A**) The mice (*n* = 5 per treatment group) were treated with cisplatin (3 mg/kg) or anti-API-5 (2.4 mg/kg). The tumor volumes after 4 weeks of treatment showed an inhibition effect of anti-API-5 peptide on both XBC-R and XBC-S growth. (**B**) The anti-API-5 peptide effect on necrosis, angiogenesis, mitosis and apoptosis in XBC-R (*n* = 5). A significant difference was found for the extent of necrosis between absence of treatment and anti-API-5 peptide treatment at 2.4 mg/kg. Concerning angiogenesis, the CD31 positive cell count decreased by half with API-5 peptide treatment. Cell counts for the proliferation of KI67 positive cells also decreased significantly, by more than half. For apoptosis, caspase-3 cleaved positive cell counts increased with anti-API-5 peptide. ^*^ *p* versus control <0.05.

At a concentration of 2.4 mg/kg, anti-API-5 peptide inhibited tumor growth in the XBC-R chemoresistant xenograft model (Figure 3A and Supplementary Figure 3). In contrast, cisplatin was not effective in inhibiting XBC-R growth.

For the XBC-S TNBC xenograft, anti-API-5 peptide also inhibited tumor growth (Figure 3B), in a range similar to that for XBC-R xenograft, since growth inhibition coefficients for anti-API-5 peptide were -0.6 and -0.8 for XBC-R and XBC-S xenografts respectively (Tables 1 and 2, Supplementary Figure 3). Associating 2.4 mg/kg of API-5 peptide with cisplatin did not improve the API-5 peptide effect on tumor growth (data not shown).

### Anti-API-5 peptide inhibited angiogenesis

In XBC-R xenografts treated with anti-API-5 peptide, we found a 5-fold increase in tumor necrosis areas at day 28 compared to untreated tumor xenografts (45% *versus* 18%, *p* < 0.01). This vascular effect of anti-API-5 peptide was associated with a 2-fold decrease in numbers of CD31-expressing murine vessels (4% in treated mice *versus* 8% in untreated tumor xenografts, *p* = 0.01, Figure 3B).

We also found a direct effect of anti-API-5 peptide on human tumor cells with proliferation inhibition in treated tumor xenografts at day 28 (30% of Ki67-expressing cells in treated mice *versus* 70% in untreated mice, *p* = 0.01). There was also a significant increase in numbers of apoptotic cells (12.5% of cleaved caspase-3-expressing tumor cells in treated mice *versus* 2.5% in untreated mice, *p* < 0.01) (Figure 3B).

For the XBC-S chemosensitive TNBC xenograft model, anti-API-5 peptide induced tumor necrosis associated with a decrease in microvessel density, but to a lesser degree than in XBC-R ([20% versus 45% of necrosis) (Supplementary Figure 5). Anti-API-5 also induced a decrease in tumor cell proliferation (by about 1.75). However, we did not find a significant increase in numbers of apoptotic tumor cells.

Overall, anti-API-5 peptide induced tumor growth inhibition in both chemoresistant and chemosensitive models, mainly through an anti-angiogenic effect.

### Regulation of API-5 expression under conditions of hypoxic and metabolic stress

**Figure 4:**
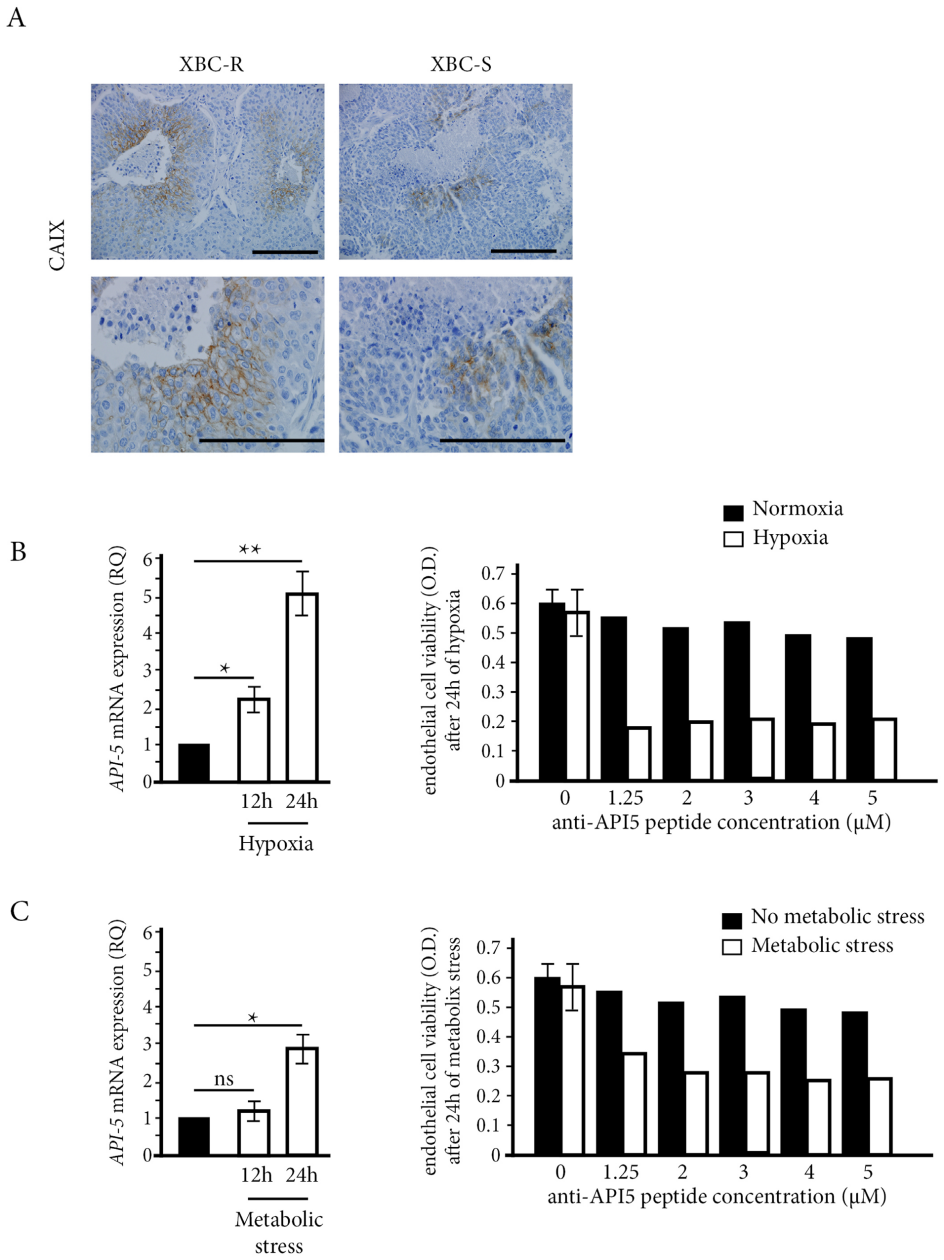
The influence of stress conditions on API-5 expression and inhibition. (**A**) Hypoxia CAIX immunostaining on XBC-R and XBC-S (*n* = 5 for each). CAIX labelling and necrotic areas were detected at a magnification of 200 (top panel) and 400 (bottom panel). (**B**, **C**) API-5 expression in HMEC cells and the effect of anti-API-5 peptide under hypoxia and metabolic stress conditions respectively. API-5 expression showed an increase after 12 h and 24 h of hypoxia and metabolic stress respectively. The effect of anti-API-5 peptide was more efficient under stress conditions on HMEC cell viability.

In untreated XBC-R and XBC-S models, we found that necrotic areas were larger in XBC-R compared to XBC-S xenografts (18% versus 7% respectively, *p* < 0.05) (Figure 4A). In addition, when we assessed the expression of carboxyanhydrase IX (CAIX), a hypoxic marker, we found a preferential distribution in peri-necrotic areas, with larger numbers of CAIX-expressing cells in XBC-R than in XBC-S (72% vs 32%, *p* < 0.05) (Figure 4A).

Then we studied the link between hypoxic and/or metabolic stress conditions and the anti-tumor effect of anti-API-5 peptide. Metabolic stress was reproduced by eliminating growth factors in culture. We first assessed the mRNA expression of *API-5* in HMEC endothelial cells cultured under conditions of hypoxic or metabolic stress for 12 h and 24 h (Figure 4B and 4C, respectively). Both stress conditions significantly increased mRNA *API-5* expression after 24 h. This was particularly true for hypoxic stress with a significant 5-fold increase compared to normoxic conditions. Using MTT assay, hypoxic or metabolic stress conditions were required to see a significant cytotoxic effect of anti-API-5 peptide on HMEC endothelial cells. Under hypoxic conditions, the inhibitory effect of anti-API-5 peptide was about 70%, while there was no effect of the peptide under normoxic conditions. Under metabolic stress conditions, the inhibitory effect of anti-API-5 peptide was about 50%, compared to no significant effect in the normoxic condition.

## DISCUSSION

API-5 is a survival protein interacting with the protein acinu, preventing its cleavage by caspase-3 and thus inhibiting apoptosis [7]. An analysis of biopsies from 78 women with locally advanced TNBC before chemotherapy showed that API-5 was more markedly expressed in tumor endothelial cells of chemoresistant tumors as opposed to non-resistant tumors. We showed that anti-API-5 peptide enhanced apoptosis in tumors by activating the caspase-3 pathway, as previously demonstrated [8]. Further to this, an anti-angiogenic effect associated with necrosis was mainly observed.

Peptides, as therapeutic agents, possess many advantages, such as small size, ease of large-scale synthesis and good biocompatibility [24–26] Some of them, like insulin for the treatment of diabetes and aflibercept for the treatment of macular degeneration, are currently used in daily clinical practice [27–29]. However, the short serum half-life of peptides and difficulties in correctly assessing their bio-distribution are at present limiting their development in humans. The chemical modification of peptides to avoid degradation or to improve penetration into tumor cells is a way of improving their bio-distribution in patients [24–29]. Thus, an anti-API-5 peptide has been engineered with an antennapedia sequence to facilitate its penetration into tumor cells [7, 8]. In a recent pre-clinical study, anti-API-5 peptide administered intraperitoneally for seven days in mice xenografted with human melanoma cancer cell lines was mainly distributed in the tumor, the liver, the kidney, and the spleen [8]. In our study, after 28 days of treatment of patient-derived TNBC xenografts with anti-API-5 peptide administered intraperitoneally, we showed direct effects on both tumor cells and endothelial cells within the tumor, with a major anti-angiogenic effect. At the highest dose of the peptide, we also observed liver toxicity, suggesting a predominance of liver metabolism of the anti-API-5 peptide.

The main strength of our pre-clinical study resides in the TNBC xenograft models that we used. We previously demonstrated that they were relevant preclinical pharmacological tools to predict treatment response in patients [20]. Indeed, we characterised 6 TNBC xenograft models for their sensitivity, resistance to various anti-cancer drugs. We finally decide to oppose the most sensitive model to the most resistant to make this preclinical study of anti-API-5 peptide for the treatment of TNBC. In contrast, tumor xenografts from cancer cell lines have widely demonstrated their inaccuracy in predicting the clinical benefit of a new drug, since less than 1% of drugs are finally approved for patients using these preclinical models [16].

Thus, to confirm the implication of API-5 in TNBC chemoresistance we used two patient-derived xenograft models with contrasting sensitivity to drugs. One model, XBC-R, was clearly resistant to the seven drugs tested, while anti-API-5 pro-apoptotic peptide was the only drug inducing an anti-tumor effect. The XBC-R model was richer in anti-apoptotic genes compared to the XBC-S chemosensitive model. In particular, API-5 expression was higher in the XBC-R model, both for mRNA and for protein, and this was true in both tumor cells and tumor endothelial cells.

In a previous study, anti-API-5 peptide was shown to penetrate the membrane of cancer cells because of the presence of API-5 partner-interacting protein [8]. The authors demonstrated that anti-API-5 peptide with a mutation in the LZ interacting domain had no effect on tumor volume [8]. In our study, higher API-5 expression in tumor endothelial cells was in line with the considerable anti-angiogenic tissue effect that we observed after 4 weeks of treatment with anti-API-5 peptide. In addition, tumor growth inhibition without a decrease in tumor volume is a hallmark of anti-angiogenic drugs [29]. For this reason, new radiological criteria combining changes in tumor radiological density and in tumor size have been implemented in daily clinical practice to more efficiently distinguish prognostic groups of patients with metastatic renal cancer treated with anti-angiogenic drugs [30, 31].

What could be the added value of a new anti-angiogenic drug? Indeed, there are several anti-angiogenic drugs currently approved for the treatment of kidney cancer, lung cancer, ovarian cancer, and hepatocellular carcinoma [32, 33]. However, for breast cancer, including the triple negative sub-type, their benefit remains controversial [34]. In our study, anti-API-5 peptide induced an anti-tumor effect in the two patient-derived TNBC xenograft models we used. In the two TNBC xenografts, anti-API-5 peptide induced necrosis with a decrease in numbers of microvessels. However, the mechanism of anti-API-5 was different in the chemosensitive model, since the peptide did not induce an increase in apoptotic tumor cells, in contrast to the chemoresistant model. It is worth noting that in the chemoresistant model, the anti-apoptotic pathway is more activated, as shown by the transcriptomic analysis.

Furthermore, we showed a possible link between hypoxic or metabolic stress and response to anti-API-5 peptide, and this was particularly true for hypoxic stress. Hypoxia is associated with drug resistance [35–38] and we have previously demonstrated a link between cancer stem-cells, hypoxic niches and resistance to sunitinib in renal cell carcinoma [36]. *In vivo*, hypoxia has been shown to induce resistance to apoptosis in human cervical cancer cell lines through a selection of p53-mutated cells. In another model of hepatocarcinoma cells, a hypoxic environment induced the expression of BCL2 homologue survival genes [37]. We also checked for the presence or absence of necrosis in the biopsies. As reported previously in the same series of patients, the presence of necrosis was significantly associated with non-pCR [22]. This is coherent with the fact that hypoxia within a tumor can increase API-5 expression, and that targeting API-5 could reverse resistance to apoptosis in the tumor.

## MATERIALS AND METHODS

### Anti-API-5 peptide and chemotherapeutic drugs

The Anti-API-5 Peptide was synthesized by Proteogenix (Strasbourg, France) and was >95% pure, as determined by HPLC and mass spectrographic analysis. The sequence of the API-5 peptide corresponds to the Leucine-Zipper subdomain of API-5 with the Antennapedia sequence included in the first 15 amino acids, as described previously (Supplementary Figure 1A). The specificity of the peptide has been described previously [7, 8]. The sequence is: RQIKIWFQNRRMKWKKAKLNAEKLKDFKIRLQYFARGLQVYIRQLRALQGKT.

All other drugs, including cisplatin, paclitaxel, epirubicin, sunitinib, dasatinib, everolimus, oxaliplatin, bevacizumab, and gemcitabine, were purchased from Sigma (France).

### Patient follow-up, biopsies and pCR

78 women with a locally-advanced ductal TNBC, prospectively enrolled in a registry and treated with neoadjuvant chemotherapy (sequential chemotherapy using 4 cycles of a dose-dense anthracycline-based regimen followed by 4 cycles of docetaxel 100 mg/m²/cycle. The characteristics for the patients (including ethical guidelines) were described by previous work [22]) at Saint-Louis-Hospital between 2005 and 2011, were included in this study.

Breast surgery was performed after neoadjuvant chemotherapy for all patients. Pathological complete response (pCR) was defined as the absence of residual disease at the time of surgery in both breasts and the axillary lymph nodes, as described in [22]. pCR was found for 20 surgical pieces (25.6%).

### *In vivo* toxicity of anti-API-5 peptide

Anti-API-5 peptide toxicity was first assessed in nude mice (*n* = 5) without any tumor. We tested four concentrations (0.8, 1.6, 2.4 and 4.8 mg/kg) of anti-API-5 peptide, This range of concentration has commonly been used *in vivo* for the pre-clinical development of therapeutic peptides [39, 40] (See Supplementary Materials and Methods).

### XBC-R and XBC-S patient-derived TNBC xenograft models

Two patient-derived xenograft models of human TNBC were used in this study (*n* = 5 for each). One model was resistant to cisplatin, paclitaxel and epirubicin. We called this TNBC xenograft model XBC-R. The other one was sensitive to these agents; we called it XBC-S.

These xenografts were from 2 metastatic biopsies of TNBC patients and were established for an earlier pilot study to personalize treatment for women with metastatic TNBC [20]. The Clinical Research Board Ethics Committee [Comité de Protection des Personnes] approved this study (CPP Ile-de-France N°13218).

The University Institute Board Ethics Committee for experimental animal studies also approved the study (#2012-15/728-0115). The ethical guidelines that were followed met the standards required by the US guidelines [41] (See Supplementary Materials and Methods).

### Transcriptomic analyses of XBC-R and XBC-S Patient-derived TNBC xenograft models

Transcriptomic analyses were performed on the two patient-derived TNBC xenografts used in this study.

Total RNA was extracted from the frozen tumor samples (*n* = 10) using an RNeasy-Mini-Kit (Qiagen, Courtaboeuf Cedex, France), quantified on NanoDrop (LabTech, Palaiseau, France) and qualified on Bio-Rad Experion™ Automated-Electrophoresis-Station (Bio-Rad, Marnes-la-Coquette, France). Transcriptomic analyses were performed using MiltenyiBiotec Microarray service (MiltenyiBiotec, Bergisch Gladbach, Germany, see Supplementary Materials and Methods).

### Gene expression quantification in XBC-R and XBC-S patient-derived TNBC xenograft models

The expression of human genes was assessed on the whole tumor using human probes, and the expression of murine genes was assessed on murine endothelial cells sorted from xenografted tumors using murine probes. All the 10 tumors were assessed.

Tumor endothelial cell sorting was performed on fresh tumor sections (*n* = 10) by dissociating them mechanically. Suspension lysates of the tumor xenografts were incubated for 30 min at 4°C with anti-mouse CD105 antibody (monoclonal rat 1/50, clone MJ7/18, BD Pharmingen, France). Unbound antibody was washed off and the cells were incubated for 15 min at 4°C with microbeads conjugated with a rat igG2a antibody (Miltenyi Biotech, France). Endothelial CD105-positive cells were sorted as recommended by the manufacturer (Miltenyi Biotech) (See Supplementary Materials and Methods).

API-5 protein expression in XBC-R and XBC-S Patient-derived TNBC xenograft models 10 sections of frozen tumors (*n* = 5 for each) were incubated in RIPA lysis buffer (25 mM Tris-HCl pH 7.6, 150 mM NaCl, 1% NP-40, 1% sodium deoxycholate, 0.1% SDS) on ice for 30 min. API-5 expression was detected by Western Blot as described in the Supplementary Materials and Methods.

*In situ* assessment of necrosis, cell proliferation, angiogenesis, apoptosis and API-5 expression.

For each case (*n* = 5 for each), analyses were performed on 10 tumor sections by two pathologists (GB, AJ) unaware of the tumor type (See Supplementary Materials and Methods). For API-5 expression in patients’ tumor biopsies and in xenografted tumors, we performed immunohistochemistry using a rabbit monoclonal antibody (ab65836, Abcam d: 1/50) which recognizes both human and murine forms of API-5. The percentage of positive cells in 100 cancer cells was determined, and results were expressed as mean ± SEM.

### Effects of stress on anti-API-5 expression in endothelial cells

To assess the effect of stress conditions on anti-API-5 peptide cytotoxicity, Human Mammary Endothelial Cells (HMEC) were experimentally cultured either under hypoxic conditions (1% pO2), or without supplementation of growth factors to produce metabolic stress. Different concentrations of API-5 (1, 2, 3, 4 and 5 µM) were assessed. For experimental hypoxia, we chose a pO2 level of 1%, as previously defined for reproducing a hypoxic environment [26].

### Statistical analyses

The statistical analyses were performed using R 2.15.2 statistical software (R-Foundation for Statistical Computing, Vienna/Austria). For the *in vitro* studies, quantitative values were compared using Student’s *t*-test [two-tailed]. A *p* value < 0.05 was considered to be significant.

## CONCLUSIONS

Our results showed that the API-5 protein could be a valid therapeutic target in chemoresistant TNBC. Methods targeting API-5 deserve further clinical development among women with metastatic TNBC.

## SUPPLEMENTARY MATERIALS



## Abbreviations

API-5: Apoptosis Inhibitor-5
pCR: pathological complete response
TNBC: Triple negative breast cancer
XBC: patient-derived TNBC xenografts
XBC-R: patient-derived TNBC xenografts resistant to tested drugs
XBC-S: patient-derived TNBC xenografts sensitive to tested drugs
