# Supplementary material for: High expression of apoptosis protein (Api-5) in chemoresistant triple-negative breast cancers: an innovative target
Source: Oncotarget. 2019 Nov 12;10(61):6577–88. doi: 10.18632/oncotarget.27312 (PMC6859922; doi:10.18632/oncotarget.27312)
Supplement: Supplementary file 1 [file oncotarget-10-6577-s001.pdf]

# High expression of apoptosis protein (Api-5) in chemoresistant triple-negative breast cancers: an innovative target

## SUPPLEMENTARY MATERIALS

## MATERIALS AND METHODS

### *In vivo* toxicity of anti-API-5 peptide

Anti-API-5 peptide was injected intraperitoneally in mice ($n$ = 5) twice a week for 28 days. For each mouse, weight and clinical stage were daily assessed. After 28 days of treatment, mice were sacrificed and all organs were formaldehyde-fixed and paraffin-embedded for assessment of tissue toxicity.

### Patient derived xenografts (PDX) of human TNBC and assessment of response to cytotoxic drugs

With their informed consent, the two patients had had imaging-guided biopsies of a metastatic site at the time of relapse, before any medical treatment. During this procedure, three tumor samples were obtained: (a) one part was formaldehyde-fixed and paraffin-embedded for histological analyses, (b) one part was immediately snap-frozen in liquid nitrogen and stored in Hôpital-Saint-Louis Tumorbank for molecular analysis, and (c) one was set aside in culture medium for xenograft. After successful engraftment, tumor growth was measured in two perpendicular diameters with a caliper. Tumor volumes were calculated as following: $V = L \times l^2 \div 2$, L being the larger diameter (length), l the smaller (width). When tumors reached a volume of 200 mm$^3$ ($n$ = 5 mice per treatment group), after 2 to 4 weeks, the mice were treated over 28 days with different regimens of chemotherapy (see Supplementary Table 1). A daily clinical score was recorded and tumor growth was measured weekly until tumor weight reached the ethically recommended limit of less than 10% of mouse weight (Directive 2010/63/EU of European Parliament and Council of 22 September 2010 on the protection of animals used for scientific purposes; Official Journal of European Union L 276/33).

For each chemotherapy tested in mice, an inhibition growth coefficient was calculated (Supplementary Figure 2), using a ratio of the slopes (a and a') of the straight lines before and after treatment. In all xenograft models, the coefficient of inhibition for a drug is calculated as (a'-a)/a, a being the slope of the curve before the start of treatment (Day 0), and a' the slope of the curve between Day 0 and Day 28 of treatment.

For one drug, if the coefficient of inhibition was negative, the tumor was considered sensitive to the drug. If, by contrast, it was positive, the tumor was considered resistant to this drug.

### Transcriptomic analyses of human tumor samples

A linear T7-based amplification step was performed from 0.5 µg of all RNA samples. To produce Cy3-labeled cRNA, the RNA samples were amplified and labeled by using an Agilent-Quick-labeling kit (Agilent Technologies, Santa Clara, CA, USA). The yields of cRNA and the dye-incorporation rate were measured with an ND-1000 Spectrophotometer (NanoDrop, LabTech). Hybridization was performed according to the Agilent 60-mer oligo-microarray processing protocol: 1.65 µg of Cy3-labeled cRNA was hybridized overnight at 65°C to Agilent-Whole-Human-Genome-Oligo-Microarrays 4x44K, and fluorescence signals were detected by using Agilent's Microarray-Scanner. Agilent-FE-Software determined feature intensities, and quantile normalization was performed with the Agi4x44PreProcess R package. Subsequent analyses were carried out with R 3.01 software (Foundation for Statistical Computing, Vienna, Austria) and based on log2 single-intensity expression data. Classification was provided by correlating gene expression profiles with the centroids for each of the six TNBC subtypes described by Lehmann and colleagues (26), https://genome.unc.edu/pubsup/breastGEO/pam50_centroids.txt).

### Gene expression quantification in tumor xenografts

For RT-qPCR, total RNA was reverse-transcribed using random primers with SuperScriptTM II-Reverse-Transcriptase (Invitrogen/Saint-Aubin/France). Each qPCR assay was performed according to the MIQE guidelines (minimum information for publication of quantitative real-time PCR experiment) [1] The qPCR reactions were performed using fluorescent probes on a CFX96 Real Time System (Bio-Rad) to determine BID, CFLAR/CFLIP, PIK3R5, API-5 gene expression levels, using Hs05102792-m1 (human BID), Hs01116280-m1 (human CFLAR/CFLIP), Hs051046353-m1 (human PIK3R5), Hs00362482_g1 (human API-5) and Mm

00599749_m1 (murin API-5) as primers (Applied-Biosystem/France). The reference gene was human TBP with the primer Hs99999910_m1 or murine Taf1a with the primer Mm00468829. A blank sample (no cDNA) was included, and experiments were performed in triplicate, with each sample in duplicate on the PCR plate. The results were expressed as 2-ΔΔCq (relative quantification).

## API-5 protein expression in human tumor xenografts

10 sections of frozen tumors ($n = 5$ for each) were incubated in 500 µL RIPA lysis buffer (25 mM Tris-HCl pH 7.6, 150 mM NaCl, 1% NP-40, 1% sodium deoxycholate, 0.1% SDS) on ice for 30 min. Cell debris of tumors were removed by centrifugation at 16,000 × g for 10 min. Protein concentration was determined by bicinchoninic acid protein assay (Thermo Fisher Scientific, Waltham, MA, USA). A 50 µg protein sample from each sample was loaded on a 10% SDS-PAGE gel, and the proteins were transferred to a nitrocellulose membrane using the iBlot dry blotting system (Invitrogen, Carlsbad, CA, USA). The membranes were blocked with 5% non-fat dry milk for 1 h and incubated with anti-API-5 (ab56392; 1:1000; Abcam) at 4°C overnight. The membranes were then washed with a Tris buffered saline with Tween 20 buffer for 1 h and incubated with the appropriate horseradish peroxidase-conjugated secondary antibodies (Invitrogen) diluted in blocking buffer, for 1 h at room temperature. Subsequent to washing, western blotting luminol reagent (Santa Cruz Biotechnology, Inc., Dallas, TX, USA) was added to the membranes and the chemioluminescence was recorded using a Fuji LAS-3000 system (Fujifilm, Tokyo, Japan). The membranes were then treated with an antibody-stripping buffer (Gene Bio-Application Ltd., Kfar Hanagid, Israel) and incubated with anti-actin antibody (1:4,000 dilution; Sigma-Aldrich) as a control.

## *In situ* assessment of necrosis, cell proliferation, angiogenesis, apoptosis and API-5 expression

When present, necrosis was delineated on virtual slides created on a Nanozoomer2.0H scanner (Hamamatsu/Japan), and quantified using DotSlide2 software. Results were expressed as the sum of necrotic areas for each section, and the mean ± SEM.

For microvessel density, proliferation and apoptotic counts, an indirect immunoperoxidase method was performed on 5 µm-thick tissue sections, using monoclonal mouse anti-human Ki67 antibody (Clone MIB-1, DakoCytomation, France) as primary antibody for proliferation, polyclonal rabbit anti human cleaved-caspase-3 antibody (Asp175, cell signaling, France) as primary antibody for apoptosis, and rabbit polyclonal anti-mouse anti-CD34 antibody (Sigma-Aldrich, France) as primary antibody for microvessel density.

To characterize hypoxic areas in the xenografted tumors, we assessed the *in situ* expression of a hypoxic-related protein CAIX [2]. We performed immunohistochemistry staining using anti-human CAIX antibody (NB100-417, Novus Biologicals, France) as primary antibody.

For API-5 expression in patients' tumor biopsies and in xenografted tumors, we performed immunohistochemistry using a rabbit monoclonal antibody (ab65836, Abcam d: 1/50) which recognizes both human and murine forms of API-5.

For the scoring, the percentage of positive cells in 100 cells was determined, and results were expressed as mean ± SEM.

As secondary antibodies, an anti-rabbit or anti-mouse OmniMap detection kit (Roche diagnostic, Meylan, France) were used. The systematic controls used were absence of primary antibody and use of an irrelevant primary antibody of the same isotype.

For each tumor section analyzed and for each marker, proliferation and apoptotic cells counts were performed on five different fields at x400 magnification, using a ProvisAX70 microscope (Olympus, Tokyo) with wide-field eyepiece number 26.5 providing a field size of 0.344mm$^2$ at X400 magnification. Microscopic pictures were captured using a ColorView III digital camera, and analyzed using Olympus-SIS Cell F software. The percentage of positive cells in 100 cancer cells was determined, and results were expressed as mean ± SEM. For microvessel density, CD34-positive microvessels were counted on ten different fields at X400 magnification. For assessment of CAIX expressing tumor cells, at magnification x250, 100 µm diameter-circles were drawn around necrotic areas. In the areas defined by each circle (corresponding to 5887 µm$^2$), CAIX positive tumor cells were counted. The number of positive cells was related to the total number of tumor cells in a given surface area studied.

Results were expressed as the mean ± standard deviation.

## *In vitro* proliferation assay in metabolic stress or hypoxic condition

Human microvascular endothelial cell (HMEC) lines (Dr TL Lawley, Department of Dermatology, Atlanta, GA, USA) were cultured under normoxic conditions (20% pO2) in MCDB-131 medium supplemented with 1 µg/mL hydrocortisone, 10 ng/mL EGF, 2 mM L-glutamine, 100 µg/mL streptomycin, 100 units/mL penicillin, and 15% fetal calf serum. HMEC cells were controlled for mycoplasma free using mycoplasma kit (Sigma, France).

Cells were exposed to increased concentrations of anti-API-5 peptide ranging from 1.25 µM to 10 µM. After 12 or 24 h incubation, cell viability was determined by the colorimetric conversion of tetrazolium MTT (3-[4. 5-dimethylthiazol-2-yl]-2.5-diphenyltetrazolium bromide; Sigma, France) into formazan, to estimate the

relative number of viable cells. Cells were incubated with 0.4 mg/ml MTT for 4 hours at 37°C. After incubation, the supernatant was discarded, and the cells suspended in 0.1 ml of DMSO. The absorbance was measured at 560 nm using a Fluostar Optima microplate reader (BMG LabTech, France). Wells with untreated cells or with drug-containing medium without cells were used as positive and negative controls respectively.

## REFERENCES


1. Johnson G, Nour AA, Nolan T, Huggett J, Bustin S. Minimum information necessary for quantitative real-time PCR experiments. Methods Mol Biol. 2014; 1160:5–17. https://doi.org/10.1007/978-1-4939-0733-5_2. [PubMed]

2. Rademakers SE, Lok J, van der Kogel AJ, Bussink J, Kaanders JH. Metabolic markers in relation to hypoxia; staining patterns and colocalization of pimonidazole, HIF-1α, CAIX, LDH-5, GLUT-1, MCT1 and MCT4. BMC Cancer. 2011; 11:167. https://doi.org/10.1186/1471-2407-11-167. [PubMed]


**Supplementary Table 1: Drug tested in xenografted mice**

| Drug | Dose (mg/kg) | Mode of administration | Number of injections/week |
|---|---|---|---|
| Cisplatin | 3 | IP | 1 |
| Paclitaxel | 20 | IP | 2 |
| Epirubicin | 1 | IP | 1 |
| Everolimus | 5 | per os | 7 |
| Oxaliplatin | 1 | IP | 2 |
| Dasatinib | 15 | per os | 7 |
| Oxaliplatin | 1 | IP | 2 |
| Sunitinib | 20 | per os | 7 |
| **Anti-API-5 peptide** | **2.4** | **IP** | **2** |

**Supplementary Table 2: Differential KEGG pathways analysis in both XBC-R and XBC-S tumor xenografts**

| Pathways | Gene count | *P* value | Genes |
|---|---|---|---|
| **Apoptosis** | **10** | **<0.05** | **BID, TNFRSF1A, PRKAR2B, CFLAR, TNFSF10, TNF, IL1B, PIK3R5, NFKB1, IL3RA** |
| Cytokine-cytokine receptor interaction | 39 | <0.01 | CXCL1, CCL3, TNF, CXCL5, IL18, KIT, CCL5, CCL4, CXCL12, CCL28, CXCL10, CCL24, LIF, TNFRSF1A, IL23A, CCL20, IL1B, TPO, PDGFC, GHR, IL6, MET, CCL19, CCL18, LEP, INHBA, VEGFC, TNFSF10, CCR6, CCL14, CXCL14, CXCL13, CXCL16, VEGFA, PDGFRB, IL3RA |
| Focal adhesion | 27 | <0.01 | CAV3, CAV2, CAV1, PGF, COL3A1, COL2A1, COMP, ITGB6, PDGFC, PIK3R5, THBS1, THBS4, FN1, COL4A2, COL4A1, TNXB, BRAF, MET, IGF1, VEGFC, LAMA4, LAMC3, VEGFA, ITGA7, COL1A2, PDGFRB, COL1A1 |
| Chemokine signaling pathway | 22 | <0.01 | CXCL1, PRKCZ, CCL3, CXCL5, BRAF, CCL19, NFKB1, CCL5, CXCL12, CCL4, CCL28, CCL18, CXCL10, CCL24, CCR6, CCL14, CXCL14, CCL20, CXCL13, CXCL16, PIK3R5, GNG4 |
| ECM-receptor interaction | 19 | <0.01 | COL4A2, COL4A1, TNXB, COL3A1, HSPG2, COL2A1, CD47, LAMA4, CD36, LAMC3, COMP, ITGA7, ITGB6, COL1A2, AGRN, COL1A1, THBS1, THBS4, FN1 |
| Toll-like receptor signaling pathway | 14 | <0.01 | CCL3, IL6, TNF, NFKB1, CCL5, CCL4, CXCL10, CD86, IRF5, MAPK13, MAP3K8, IL1B, PIK3R5, IRF3 |
| Cell adhesion molecules (CAMs) | 13 | <0.05 | PVR, HLA-DQB1, SELP, CLDN4, CLDN3, CLDN5, HLA-C, CDH2, NCAM1, CD86, CD34, PVRL2, PECAM1 |
| NOD-like receptor signaling pathway | 10 | <0.01 | CXCL1, NOD2, IL6, TNF, MEFV, MAPK13, IL18, IL1B, NFKB1, CCL5 |
| Cytosolic DNA-sensing pathway | 9 | <0.01 | IL6, IL18, RIPK3, IL1B, IRF3, NFKB1, CCL5, CCL4, CXCL10 |
| PPAR signaling pathway | 9 | <0.05 | CPT1C, LPL, CD36, PPARG, FABP4, AQP7, ADIPOQ, ACSL6, ANGPTL4 |
| Cysteine and methionine metabolism | 6 | <0.05 | ADI1, SRM, MTR, MTAP, CDO1, CBS |

A

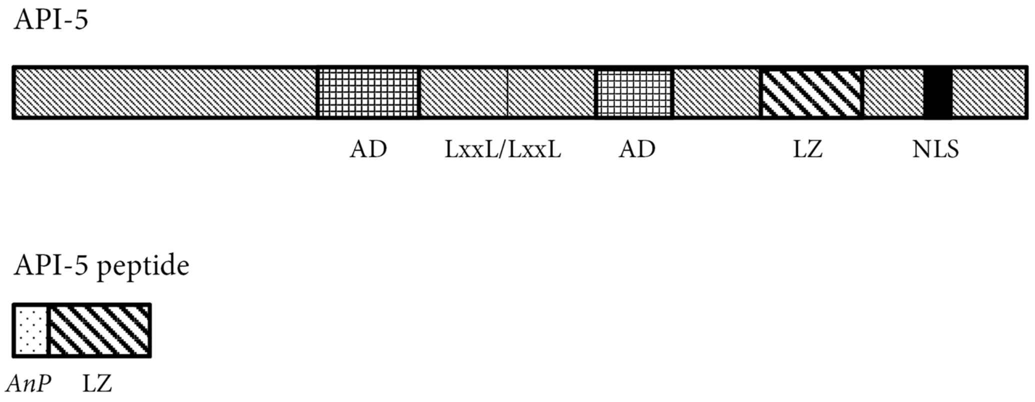


B

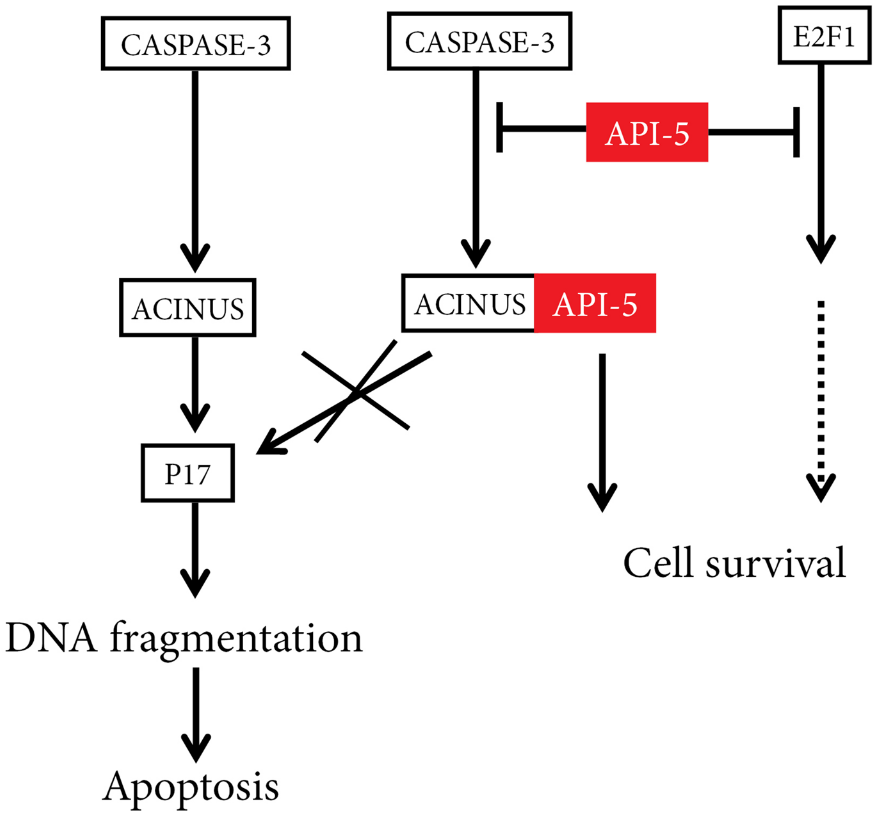


**Supplementary Figure 1:** (**A**) API-5 has two acid domains (AD), followed by hydrophobic repeated sequences (LxxL), a leucin zipper domain (LZ) and a nuclear localisation (NLS). API-5 peptide contains the antennapedia penetrating domain (AP) and the highly conserved LZ domain. (**B**) The anti-apoptotic effect of API-5 on tumor cell apoptosis. API-5 suppresses apoptosis in drugs inducing apoptosis by interacting with acinus and preventing its cleavage by caspase-3, and consequently the generation of an active P17 fragment to induce DNA fragmentation. Also API-5 inhibits the apoptosis induced by E2 promoter-binding factor (E2F) in multiple cell types and developmental context.

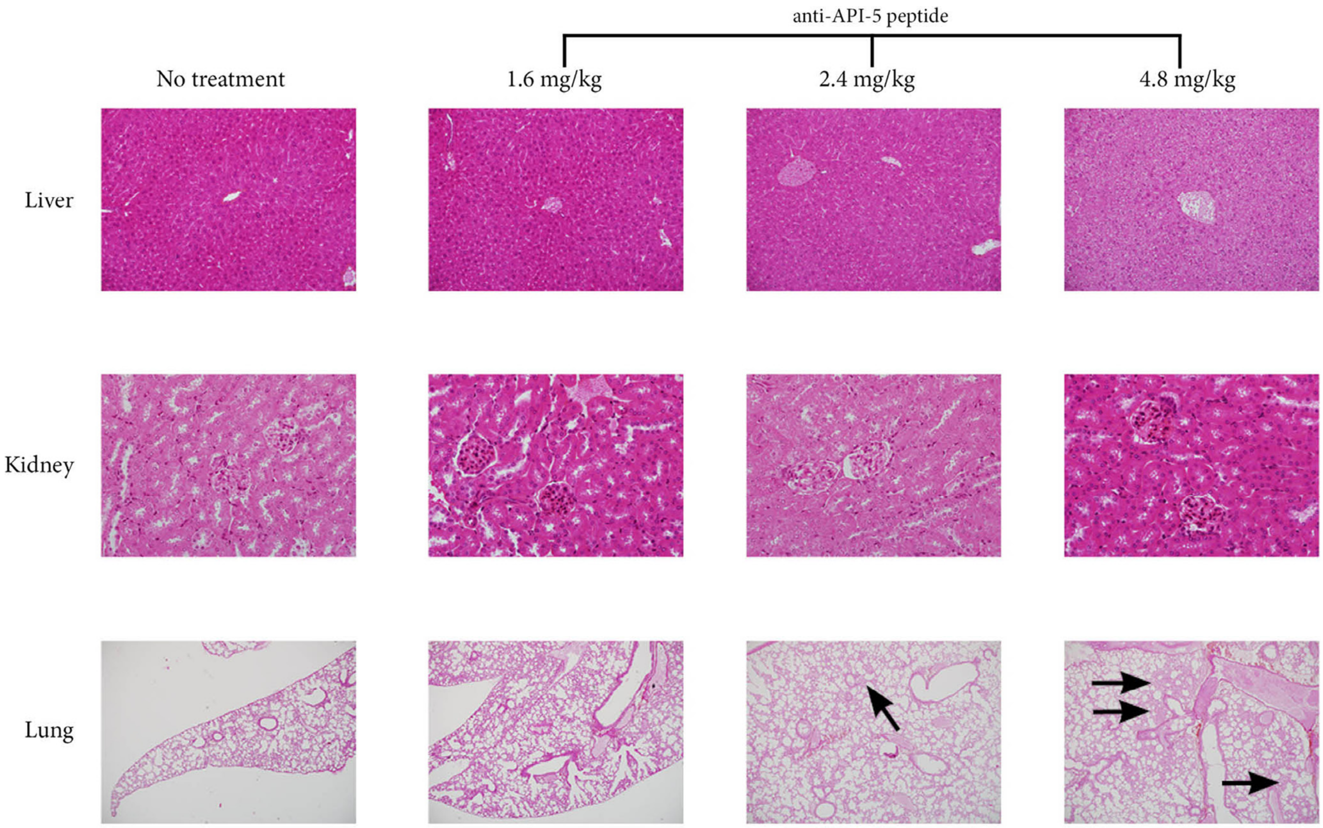


**Supplementary Figure 2: Histological analysis of toxicity studies of the anti-API-5 peptide: Concentrations of anti-API-5 peptide ranged from 1.6 mg/kg to 4.8 mg/kg.** The peptide was injected i.p twice a week for 28 days. Then the mice were euthanised and dissected. Liver, kidneys, and lung were formaldehyde-fixed and embedded in paraffin and hematoxylin-eosin stained for tissue analysis. Diffuse hepatocyte cytoplasmic clarification and several lung edemas (arrows) were particularly found in mice treated at 4.6 mg/kg.

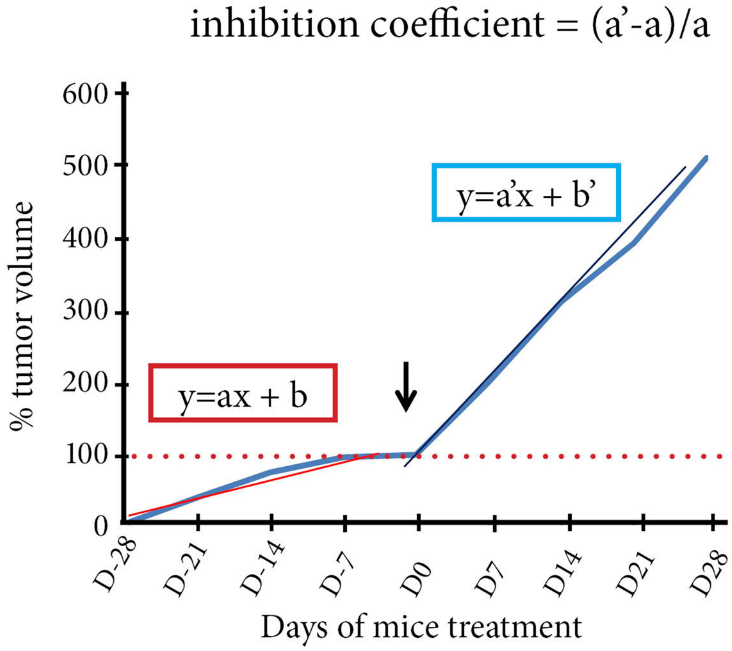


**Supplementary Figure 3: Growth inhibition coefficient of XBC-S and XBC-R.** In all xenograft models, the coefficient of inhibition for a drug is calculated as (a'-a)/a, a being the slope of the curve before the start of treatment (Day 0), and a' the slope of the curve between Day 0 and Day 28 of treatment. If this inhibition growth coefficient was found to be less than 0, the tumor was considered sensitive to the chemotherapy administered, if it was above 0, the tumor was considered resistant to this chemotherapy.

Apoptosis pathway

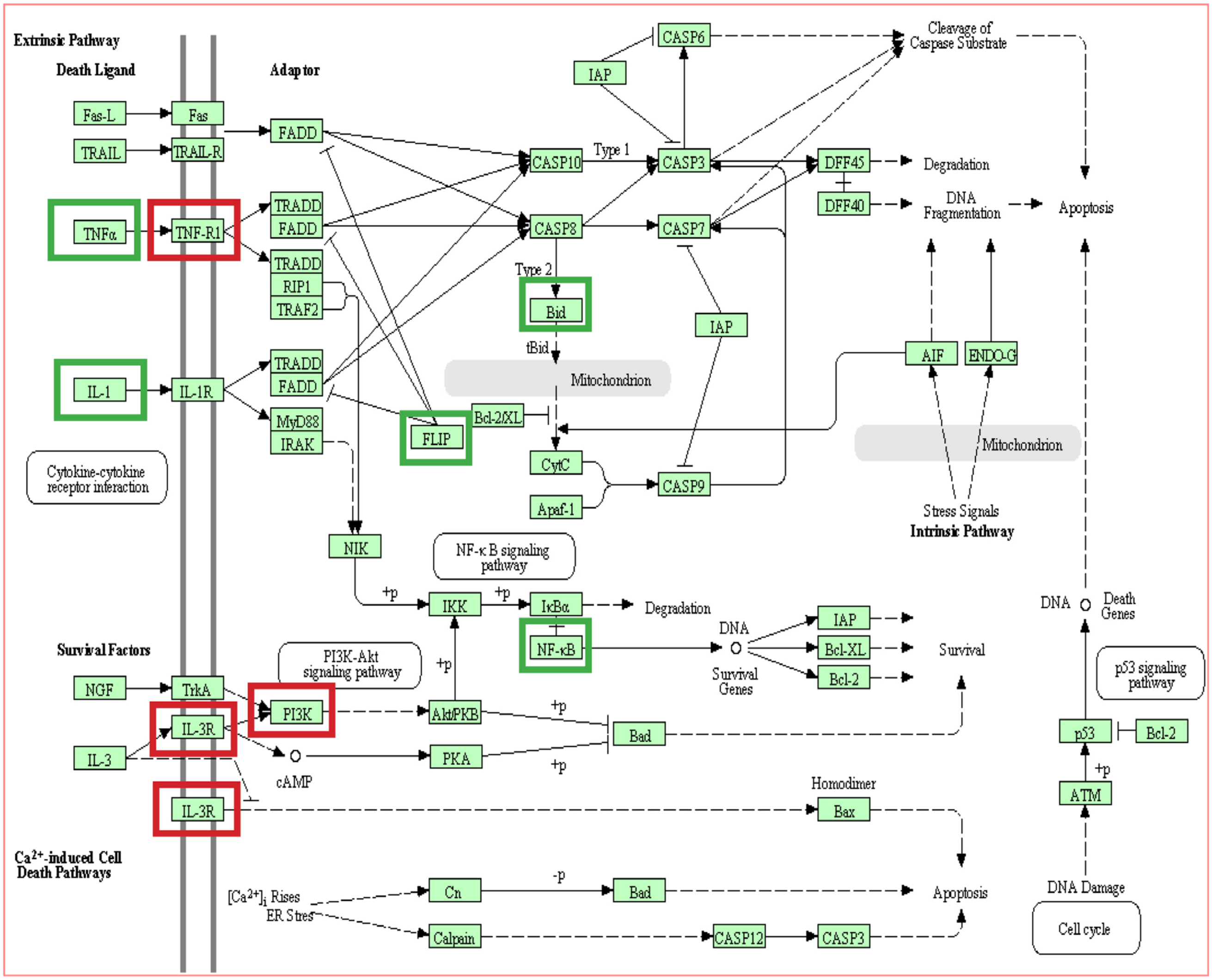


**Supplementary Figure 4: Differential apoptotic pathways obtained from transcriptomic analysis of resistant and sensitive TNBC.** The apoptosis pathway was obtained from combined data bank http://www.genome.jp/dbget-bin/www_bget?hsa04210. The red boxes show overexpression of genes in resistant TNBC. The green boxes show overexpression of genes in sensitive TNBC.

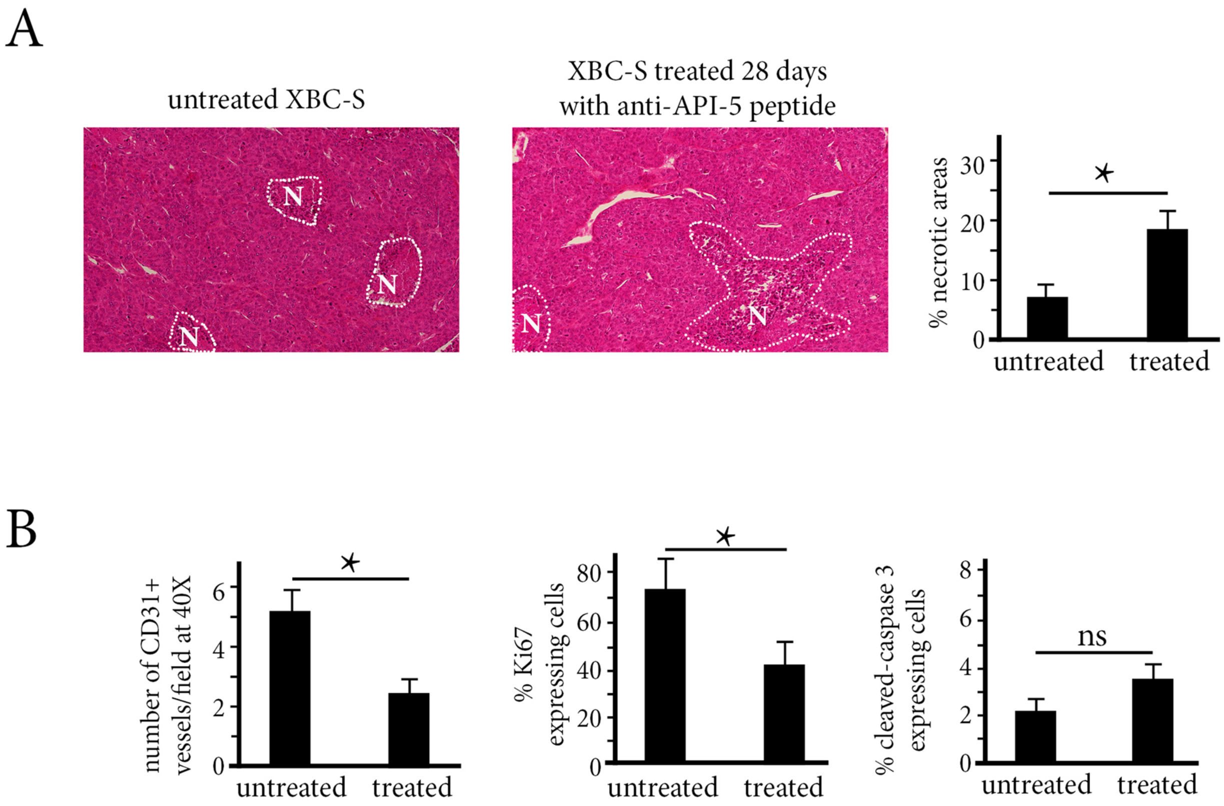


**Supplementary Figure 5: Anti-API-5 effect on necrosis, angiogenesis, mitosis and apoptosis in XBC-S.** Histological analyses of XBC-S. (**A**) For the extent of necrosis a significant 2-fold increase is observed after anti-API-5 peptide treatment at 2.4 mg/kg. (**B**) For angiogenesis, CD31 positive cell counts fall by nearly a half with API-5 peptide treatment. For proliferation, KI67-positive cell counts also decrease significantly by around half. For apoptosis caspase-3 cleaved positive cell counts no significant effect is observed between control and anti API-5 peptide treated cells (*p* versus control>0.05); $^{*}p$ versus control < 0.05.
